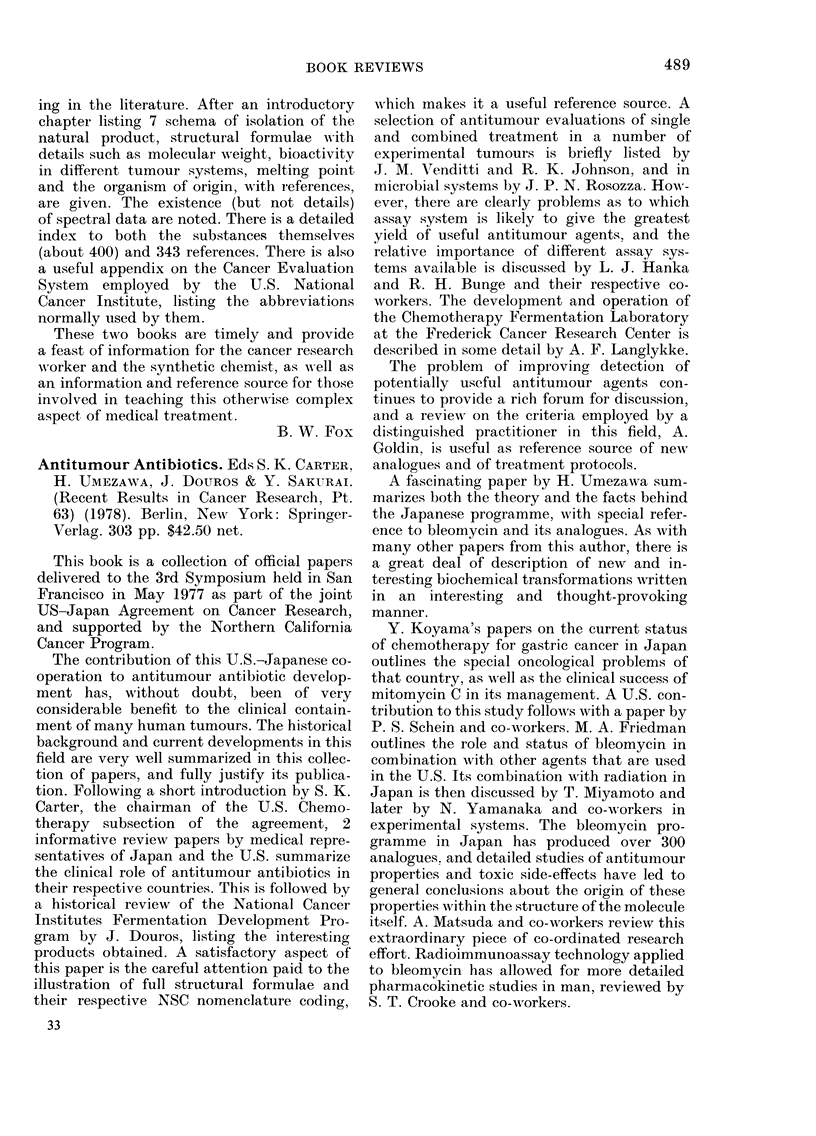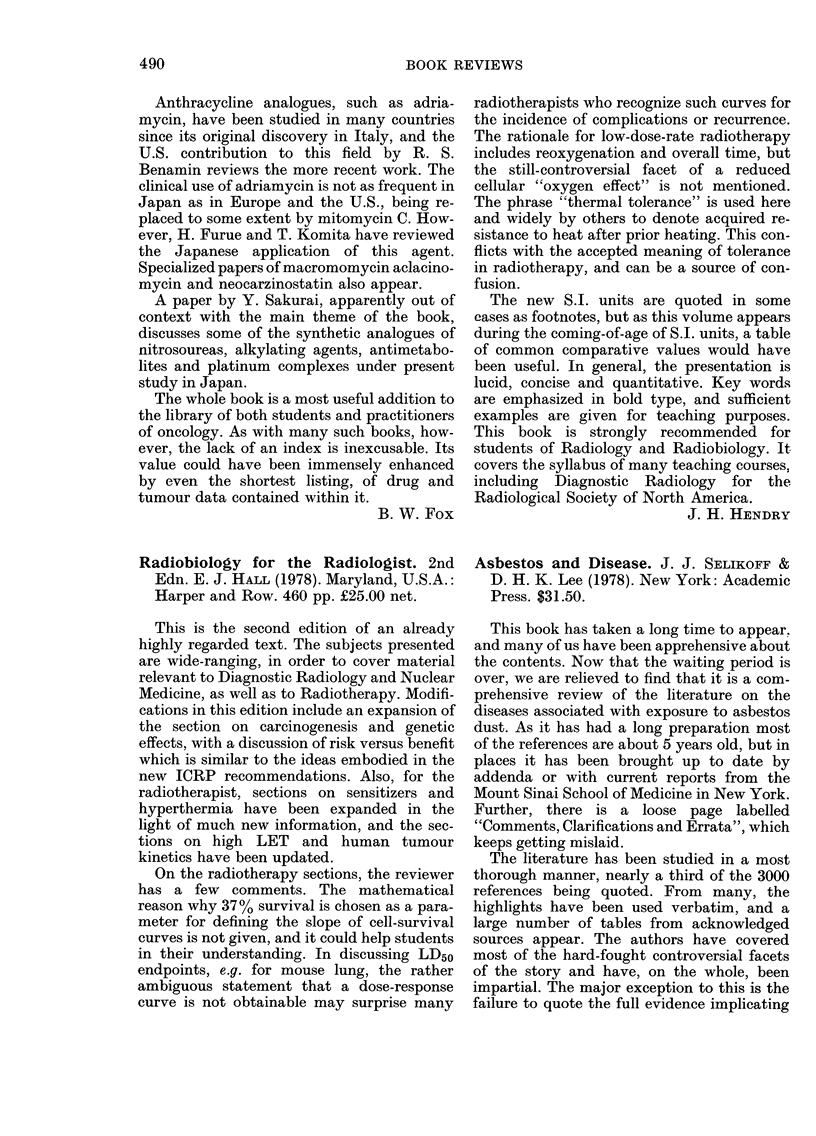# Antitumour Antibiotics

**Published:** 1979-04

**Authors:** B. W. Fox


B. W. Fox

**Antitumour Antibiotics.** Eds S. K. Carter, H. Umezawa, J. Douros & Y. Sakurai. (Recent Results in Cancer Research, Pt. 63) (1978). Berlin, New York: Springer-Verlag. 303 pp. $42.50 net.

This book is a collection of official papers delivered to the 3rd Symposium held in San Francisco in May 1977 as part of the joint US–Japan Agreement on Cancer Research, and supported by the Northern California Cancer Program.

The contribution of this U.S.–Japanese co-operation to antitumour antibiotic development has, without doubt, been of very considerable benefit to the clinical containment of many human tumours. The historical background and current developments in this field are very well summarized in this collection of papers, and fully justify its publication. Following a short introduction by S. K. Carter, the chairman of the U.S. Chemotherapy subsection of the agreement, 2 informative review papers by medical representatives of Japan and the U.S. summarize the clinical role of antitumour antibiotics in their respective countries. This is followed by a historical review of the National Cancer Institutes Fermentation Development Program by J. Douros, listing the interesting products obtained. A satisfactory aspect of this paper is the careful attention paid to the illustration of full structural formulae and their respective NSC nomenclature coding, which makes it a useful reference source. A selection of antitumour evaluations of single and combined treatment in a number of experimental tumours is briefly listed by J. M. Venditti and R. K. Johnson, and in microbial systems by J. P. N. Rosozza. However, there are clearly problems as to which assay system is likely to give the greatest yield of useful antitumour agents, and the relative importance of different assay systems available is discussed by L. J. Hanka and R. H. Bunge and their respective co-workers. The development and operation of the Chemotherapy Fermentation Laboratory at the Frederick Cancer Research Center is described in some detail by A. F. Langlykke.

The problem of improving detection of potentially useful antitumour agents continues to provide a rich forum for discussion, and a review on the criteria employed by a distinguished practitioner in this field, A. Goldin, is useful as reference source of new analogues and of treatment protocols.

A fascinating paper by H. Umezawa summarizes both the theory and the facts behind the Japanese programme, with special reference to bleomycin and its analogues. As with many other papers from this author, there is a great deal of description of new and interesting biochemical transformations written in an interesting and thought-provoking manner.

Y. Koyama's papers on the current status of chemotherapy for gastric cancer in Japan outlines the special oncological problems of that country, as well as the clinical success of mitomycin C in its management. A U.S. contribution to this study follows with a paper by P. S. Schein and co-workers. M. A. Friedman outlines the role and status of bleomycin in combination with other agents that are used in the U.S. Its combination with radiation in Japan is then discussed by T. Miyamoto and later by N. Yamanaka and co-workers in experimental systems. The bleomycin programme in Japan has produced over 300 analogues, and detailed studies of antitumour properties and toxic side-effects have led to general conclusions about the origin of these properties within the structure of the molecule itself. A. Matsuda and co-workers review this extraordinary piece of co-ordinated research effort. Radioimmunoassay technology applied to bleomycin has allowed for more detailed pharmacokinetic studies in man, reviewed by S. T. Crooke and co-workers.

Anthracycline analogues, such as adriamycin, have been studied in many countries since its original discovery in Italy, and the U.S. contribution to this field by R. S. Benamin reviews the more recent work. The clinical use of adriamycin is not as frequent in Japan as in Europe and the U.S., being replaced to some extent by mitomycin C. However, H. Furue and T. Komita have reviewed the Japanese application of this agent. Specialized papers of macromomycin aclacinomycin and neocarzinostatin also appear.

A paper by Y. Sakurai, apparently out of context with the main theme of the book, discusses some of the synthetic analogues of nitrosoureas, alkylating agents, antimetabolites and platinum complexes under present study in Japan.

The whole book is a most useful addition to the library of both students and practitioners of oncology. As with many such books, however, the lack of an index is inexcusable. Its value could have been immensely enhanced by even the shortest listing, of drug and tumour data contained within it.

B. W. Fox